# Superoxide dismutase 2 ameliorates mitochondrial dysfunction in skin fibroblasts of Leber’s hereditary optic neuropathy patients

**DOI:** 10.3389/fnins.2022.917348

**Published:** 2022-08-09

**Authors:** Qingru Zhou, Shun Yao, Mingzhu Yang, Qingge Guo, Ya Li, Lei Li, Bo Lei

**Affiliations:** ^1^Henan Provincial People’s Hospital, Zhengzhou University People’s Hospital, Zhengzhou, China; ^2^Henan Eye Hospital, Henan Provincial People’s Hospital, Henan Eye Institute, Zhengzhou, China; ^3^Xinxiang Medical University, Xinxiang, China

**Keywords:** SOD2, LHON, mitochondrial dysfunction, oxidative stress, retinal ganglion cell

## Abstract

**Background:**

In Leber’s hereditary optic neuropathy (LHON), mtDNA mutations mediate mitochondrial dysfunction and apoptosis of retinal ganglion cells. Mitochondrial superoxide dismutase 2 (SOD2) is a crucial antioxidase against reactive oxygen species (ROS). This study aims to investigate whether SOD2 could ameliorate mtDNA mutation mediated mitochondrial dysfunction in skin fibroblasts of LHON patients and explore the underlying mechanisms.

**Methods:**

The skin of normal healthy subjects and severe LHON patients harboring m.11778G > A mutation was taken to prepare immortalized skin fibroblast cell lines (control-iFB and LHON-iFB). LHON-iFB cells were transfected with SOD2 plasmid or negative control plasmid, respectively. In addition, human neuroblastoma SH-SY5Y cells and human primary retinal pigmental epithelium (hRPE) cells were stimulated by H_2_O_2_ after gene transfection. The oxygen consumption rate (OCR) was measured with a Seahorse extracellular flux analyzer. The level of ATP production, mitochondrial membrane potential, ROS and malondialdehyde (MDA) were measured separately with the corresponding assay kits. The expression level of SOD2, inflammatory cytokines and p-IκBα/IκBα was evaluated by western-blot. Assessment of apoptosis was performed by TUNEL assay.

**Results:**

LHON-iFB exhibited lower OCR, ATP production, mitochondrial membrane potential but higher level of ROS and MDA than control-iFB. Western-blot revealed a significantly increased expression of IL-6 and p-IκBα/IκBα in LHON-iFB. Compared with the negative control, SOD2 overexpression increased OCR, ATP production and elevated mitochondrial membrane potential, but impaired ROS and MDA production. Besides, western-blot demonstrated exogenous SOD2 reduced the protein level of IL-6 and p-IκBα/IκBα. TUNEL assays suggested SOD2 inhibited cells apoptosis. Analogously, in SH-SY5Y and hRPE cells, SOD2 overexpression increased ATP production and mitochondrial membrane potential, but decreased ROS, MDA levels and suppressed apoptosis.

**Conclusion:**

SOD2 upregulation inhibited cells apoptosis through ameliorating mitochondrial dysfunction and reducing NF-κB associated inflammatory response. This study further support exogenous SOD2 may be a promising therapy for the treatment of LHON.

## Introduction

With a prevalence of about 1/30,000, Leber’s hereditary optic neuropathy (LHON) is a maternally inherited disease characterized by irreversible loss of vision and atrophy of the optic nerve (Yu-Wai-Man et al., 2011). The visual prognosis is usually poor and causes legal blindness. At present, it is still an incurable condition due to the lack of effective treatments. Three mutations (MTND1-3460, MTND4-11778, and MTND6-14484) account for 95% of European cases and MTND4-11778 alone for 80% of Japanese cases (Yen et al., 2006). These mutations change the function of mitochondrial respiratory chain complex I and lead to bioenergetic defect (Lopez Sanchez et al., 2020).

Mitochondrion contains the key structure of energy production, involved in both energy metabolism and free radical metabolism. By generating almost 90% of the total amount of cellular ROS, it is the major source of ROS under normal physiological conditions (Balaban et al., 2005; Sarewicz et al., 2010; Kausar et al., 2018). ROS is produced as a result of electronic leakage from the mitochondrial respiratory chain during oxidative phosphorylation (OXPHOS) and is responsible for oxidative damage mediated by permanently modifying proteins, lipids, and mtDNA (Islam, 2017; Schofield and Schafer, 2021). In LHON, abnormal function and structure of mitochondrial respiratory chain complex I generates excessive ROS (Carelli et al., 2009; Hayashi and Cortopassi, 2015; Lopez Sanchez et al., 2016). The accumulation of ROS plays an important role in the development of LHON and other neurodegenerative diseases (Singh et al., 2019; Liskova et al., 2021).

By transforming superoxide radicals into hydrogen peroxide and oxygen, superoxide dismutase (SOD) enzymes are among the most important defense systems against oxygen radicals. Three isoforms of SOD exist in mammals, including cytoplasmic superoxide dismutase (SOD1), mitochondrial superoxide dismutase (SOD2) and extracellular superoxide dismutase (SOD3) (Sheng et al., 2014; Rosa et al., 2021). Since mitochondria are involved in the pathogenesis of LHON, we verified the hypothesis that SOD2 might protect against mitochondrial dysfunction by inhibiting oxidative stress and prevent cell death. Free radicals and other oxidants are critical determinants of the cellular signaling pathways involved in the pathogenesis of several human diseases including inflammatory diseases. SOD plays an essential pathogenic role in the inflammatory diseases by not only catalyzing the conversion of the superoxide to hydrogen peroxide and oxygen but also affecting immune responses (Hernandez-Saavedra et al., 2005; Kwon et al., 2012; Nguyen et al., 2020). Thus, we detected inflammatory cytokine expression and explored the anti-inflammatory effect in LHON is mediated by NF-κB signaling. Because it is impossible to obtain retinal ganglion cells from the patients, we constructed fibroblastic cells derived from patients’ skin tissues, which have recently been used to explore the mechanisms and therapeutic effects by other groups (Chao de la Barca et al., 2016; Morvan and Demidem, 2018; Zhou et al., 2020). To further confirm the results, additional tests were performed in human neuroblastoma SH-SY5Y cells and in human primary retinal pigmental epithelium (hRPE) cells.

## Materials and methods

### Subjects and cell culture

This study was conducted followed the Declaration of Helsinki. All protocols in this study were approved by Ethic Committees of Henan Eye Hospital [IRB approval number: HNEECKY-2019 (12)]. Informed consents were obtained from all participants. Two genetically unrelated Chinese LHON subjects carrying mutation m.11778G > A, aged 28 and 31 years old, respectively, and an age-matched (30 years old) healthy control subject without mtDNA mutation were recruited at Henan Eye Hospital. One subject carried only one mutation m.11778G > A, and the other carried multiple variants LHON associated mutations including m.11778G > A, m.10398A > G and m.4833A > G. All patients presented typical clinical manifestations of LHON. The healthy control subject did not have any ophthalmic condition and was excluded for any other diseases. The immortalized fibroblast cell lines (LHON-iFB and control-iFB) were conducted as following. The volume of 2 mm^3^ skin tissues were obtained from volunteers and soak in 75% alcohol for 2 min. The tissues were cut into pieces and washed repeatedly, followed by incubating in complete medium at 37°C for 30–60 min. A small piece was clamped into a culture flask and cultured with 2 ml complete fibroblast medium in a 5% CO_2_ incubator for 2 h. The medium was changed every 3 days until the tissue blocks grow around it. After removing tissue pieces, the cells were trypsinized and incubated with complete medium for 1 week. Cells were infected with SV40-overexpressing lentivirus (EF1α-SV40-IRES-puromycin) and puromycin was used for selection until the cells were tolerant. The successfully screened cells were expanded and subjected to experiments. The complete fibroblast medium contains DMEM/F12 medium supplemented with 10% fetal bovine serum, 10 ng/ml basic fibroblast growth factor (Labsystech, Shanghai, China) and 1% penicillin and streptomycin (Solarbio, Shanghai, China).

Primary human RPE (hRPE) cells were prepared from donor eyes from the Henan Eye Bank based on our previous protocols (Fu et al., 2017; Qiu et al., 2021; Jin et al., 2022). None of the donors had a history of eye diseases. SH-SY5Y cells were purchased from the American Type Culture Collection (ATCC, Manassas, VA, United States). The cells were cultured with DMEM medium (Invitrogen, Carlsbad, CA, United States) supplemented with 10% fetal bovine serum (Corning, Tewksbury, MA, United States), 100 U/ml penicillin and 100 U/ml streptomycin (Solarbio) in 37°C incubator.

### Plasmids and transfection

The CDS (coding sequence) of the human SOD2 gene with a flag tag at the C-terminal was synthesized and subcloned into the pcDNA3.1 (+) expression vector (Invitrogen). All plasmids were confirmed by DNA sequencing and WB analysis. When cells grow to 80% confluence, transfection was performed using Lipofectamine 3000 (Invitrogen). After transfection, the cells were harvested at 48 h for protein extraction and further analysis.

### Measurements of oxygen consumption

The rates of oxygen consumption (OCR) in fibroblast cell lines were measured with a Seahorse Bioscience XFe96 extracellular flux analyzer (Agilent, Santa Clara, CA, United States) (Dranka et al., 2011). Fibroblast cells were seeded at a density of 2.5 × 10^4^ cells per well on the polystyrene tissue culture plates. Inhibitors were added with the following concentrations: oligomycin (1.5 μM), carbonyl cyanide 4-(trifluoromethoxy) phenylhydrazone (FCCP; 1 μM), antimycin A and rotenone (0.5 μM).

### Determination of mitochondrial membrane potential

Mitochondrial membrane potential (MMP) was detected with a JC-1 Staining Kit (Beyotime, Shanghai, China). Skin fibroblast cells, hRPE cells and SH-SY5Y cells were cultured in 12-well plates separately. After treatments, cells were washed with PBS (phosphate buffer saline) and incubated with JC-1 staining fluid in darkness at 37°C for 30 min. Then, the cells were washed with cold staining buffer. Fluorescent cells were visualized using an Olympus confocal microscope. Cellular mitochondria with normal MMP emitted red fluorescence (J-aggregate), while those with abnormal MMP showed green fluorescence (J-monomer). Fluorescence intensity was quantified using Image-J software (National Institutes of Health, Bethesda, MD, United States). The MMP was reflected by ratio of red/green fluorescence intensity.

### Reactive oxygen species level detection

Skin fibroblast, hRPE and SH-SY5Y cells were subjected to reactive oxygen species (ROS) assay, respectively, according to the manufacturer’s instruction (Beyotime). Briefly, cells were incubated with corresponding serum-free medium containing with DCFH-DA at 37°C for 30 min. DCFH-DA was removed and washed with serum-free medium for three times. Fluorescence of DCF was detected using fluorescence microscope analysis (Olympus, TKY, Japan). The average optical density of each group was measured with Image-J software.

### Adenosine triphosphate and malondialdehyde assay

The ATP levels in various cells were determined by the Enhanced ATP Assay Kit (Beyotime) according to the manufacturer’s instructions. The luminescence was read on a microplate reader (Bio-Tek, Winooski, VT, United States) and the content of ATP was calculated according to ATP standard curve. ATP levels were normalized to μmol/mg protein. The lipid peroxidation levels of various cells were examined using a Lipid Peroxidation MDA Assay kit (Beyotime). The preparation of thiobarbituric acid (TBA) stock solution and MDA working solution, as well as the dilution of the standard substances were all performed in accordance with the product manual. The MDA content was measured at 532 nm using a microplate reader (Bio Tek). ATP levels and MDA levels were normalized to mmol/mg protein.

### Terminal-deoxynucleoitidyl transferase mediated nick end labeling assay

Cell apoptosis was determined by using the One-step TUNEL cell apoptosis detection kits (Beyotime) following manufacturer’s protocols. Various cells were seeded in 12-well plates separately. After treatments, cells were fixed with 4% paraformaldehyde for 30 min and washed twice using PBS. Cells were permeabilized with PBS containing 0.3% Triton X-100 at room temperature for 5 min and washed twice with PBS. A total of 50 μl TUNEL detection solution was added to the sample and incubated in the darkness at 37°C for 60 min. Every sample was photographed by a fluorescent microscope.

### Extraction of mitochondrial proteins

Mitochondrial proteins were extracted by the Cell Mitochondria Isolation kit (Beyotime). Cells were collected and resuspended in 2.5 ml of mitochondrial separation reagent containing 1 mM phenylmethylsulfonyl fluoride (PMSF). After incubation on ice for 15 min, the cell resuspension was homogenized by a glass homogenizer and centrifuged at 600 g for 10 min at 4°C. The supernatant was centrifuged at 11,000 g for 10 min at 4°C. The supernatant was discarded, whereas the precipitate containing the mitochondria was washed and re-suspended in 100 μl lysis solution with 1 mM PMSF. The lysed solution was centrifuged again and the supernatant containing mitochondrial proteins was collected for western blotting.

### Western blotting analysis

Skin fibroblast cells were washed with PBS (Solarbio) for three times and lysed with RIPA lysis buffer for WB/IP assays (Yesen, Shanghai, China) containing 1% pro-tease inhibitor cocktail (ApexBio, Housto, TX, United States) on the ice for 30 min. The supernatants were collected after centrifuging at 12,000 rpm for 15 min. The protein concentration was detected by using a bicinchoninic acid (BCA) protein kit (Beyotime). All samples were diluted with 5 × SDS loading buffer (EpiZyme, Shanghai, China) and boiled at 95°C for 5 min. Equal amounts of total protein were separated on a 10% SDS-polyacrylamide gel and transferred to polyvinylidene difluoride (PVDF) membranes (Millipore, Billerica, MA, United States). After blocking with 5% non-fat milk for 1.5 h, the membranes were incubated with specific primary antibodies against flag (1:1,000, Abcam, Cambridge, United Kingdom), p-IκB-α (1:500, Abcam), IκB-α (1:1,000, Abcam), IL-6 (1:500, Abcam), and β-actin (1:1,000, Abcam) overnight at 4°C. After washed with TBST (containing 1% Tween-20, Solarbio) for three times, the membranes were incubated with secondary antibody (1:10,000, Millipore) at room temperature for 1.5 h. The membranes were washed with TBST (containing 1% Tween-20) for three times. Signals were developed with ECL kit (Millipore), and band densitometry was performed using the AlphaView SA Software (ProteinSimple, San Jose, CA, United States). β-Actin was used as loading control. Measurements were repeated three times for each experiment. Band intensity was analyzed using the Image-J software (Fu et al., 2017; Lei et al., 2017; Qiu et al., 2021).

### Statistical Analysis

Statistical analysis was performed by using the GraphPad Prism 7 (GraphPad Software, San Jose, CA, United States). Experimental data were analyzed by Student’s *t*-test or one-way analysis of variance (ANOVA) followed by Bonferroni correction. *P*-value less than 0.05 was considered statistically significant. All data are presented as the mean ± standard deviation.

## Results

### Leber’s hereditary optic neuropathy-fibroblasts presented mitochondrial dysfunction, oxidative stress, inflammatory response, and increased apoptosis

LHON-WJ was a 28-year-old male carrying mutation m.11778G > A. His BCVA was 0.25 in the right and 0.20 in the left eye. LHON-MZT, aged 31 years old, had BCVA of 0.02 in both eyes and carried LHON associated mutations m.11778G > A, m.10398A > G, and m.4833A > G. SS-OCT revealed the retinal nerve fiber layer decreased in both cases.

**FIGURE 1:**
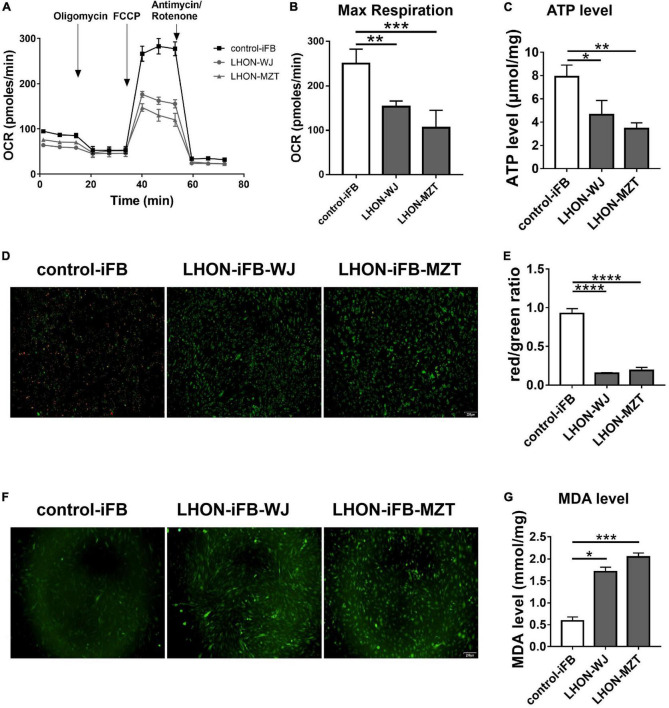
LHON-iFBs presented mitochondrial dysfunction and oxidative stress. **(A,B)** OCR, **(C)** ATP level, **(D,E)** mitochondrial membrane potential, and **(F)** ROS level in LHON-iFBs and control-iFB were measured immediately after samples were prepared from each group. **(G)** The level of MDA in fibroblasts were detected, respectively. The results (mean ± SD) were statistically significant (*p < 0.05, **p < 0.01, ***p < 0.001, ****p < 0.0001, *n* = 3–5) by using one-way ANOVA with Bonferroni correction, and the assays were performed three times.

As measured by the Seahorse XFe96 extracellular flux analyzer and microplate reader, LHON-iFBs from both patients had considerably lower OCR, particularly for maximal respiration (Figures 1A,B, *n* = 3), and ATP level (Figure 1C, *n* = 4) as compared to control-iFB. JC-1 fluorescence images and quantitative analysis revealed that LHON-iFBs displayed higher green fluorescence intensity, but much weaker red fluorescence intensity than the control-iFB, indicating a significant drop of mitochondrial membrane potential (Figures 1D,E, *n* = 4). These results indicated mitochondrial dysfunction in both LHON-iFBs.

ROS and MDA measurements were performed in LHON-iFBs to assess oxidative stress. ROS level was detected by the DCFH-DA fluorescent probe. The averaged fluorescent intensity in the LHON fibroblast cell lines was higher than that in control fibroblasts (Figure 1F, *n* = 4), indicating overproduction of ROS in LHON fibroblasts. In addition, MDA, a product of lipid peroxidation, in LHON-iFBs were higher than in control-iFB (Figure 1G, *n* = 5). These results suggested that LHON-iFBs were at a higher status of oxidative stress.

**FIGURE 2:**
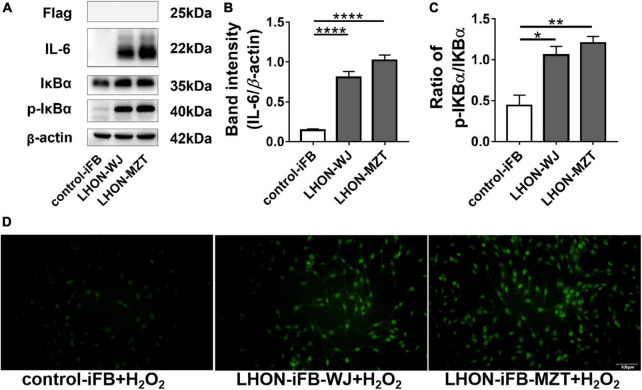
LHON-iFBs presented inflammatory response and increased apoptosis. Western blot showed that **(A,B)** protein levels of IL-6 and **(A,C)** the ratio of p-IκBα to IκBα were upregulated robustly in LHON-iFB compared to control-iFB. **(D)** TUNEL assay was performed in LHON-iFBs and control-iFB treated with same concentration of hydrogen peroxide for 12 h. The results (mean ± SD) were statistically significant (*p < 0.05, **p < 0.01, ****p < 0.0001, *n* = 3–4) by using one-way ANOVA with Bonferroni correction, and the assays were performed three times.

Western blot revealed that the ratio of p-IκBα to IκBα protein, and the expression of IL-6 increased significantly in LHON-iFBs compared to the control-iFB, indicating that phosphorylation of IκBα and the secretion of the inflammatory cytokine were upregulated in LHON-iFBs (Figures 2A–C, *n* = 4). The fibroblasts from LHON patients presented higher inflammatory response.

To explore the apoptosis status in LHON-iFBs, we performed the TUNEL assay. Hydrogen peroxide treatment was used to induce apoptosis before measurements (Miyoshi et al., 2006; Cerella et al., 2009; Fan et al., 2020). TUNEL-positive cells (indicating apoptosis) were much more in LHON-iFBs as compared with control-iFB (Figure 2D, *n* = 3), demonstrating cells bearing mtDNA mutation were more susceptible to the insult.

### Exogenous superoxide dismutase 2 protected Leber’s hereditary optic neuropathy-fibroblasts by ameliorating oxidative stress, mitochondrial dysfunction, inflammatory response and apoptosis

**FIGURE 3:**
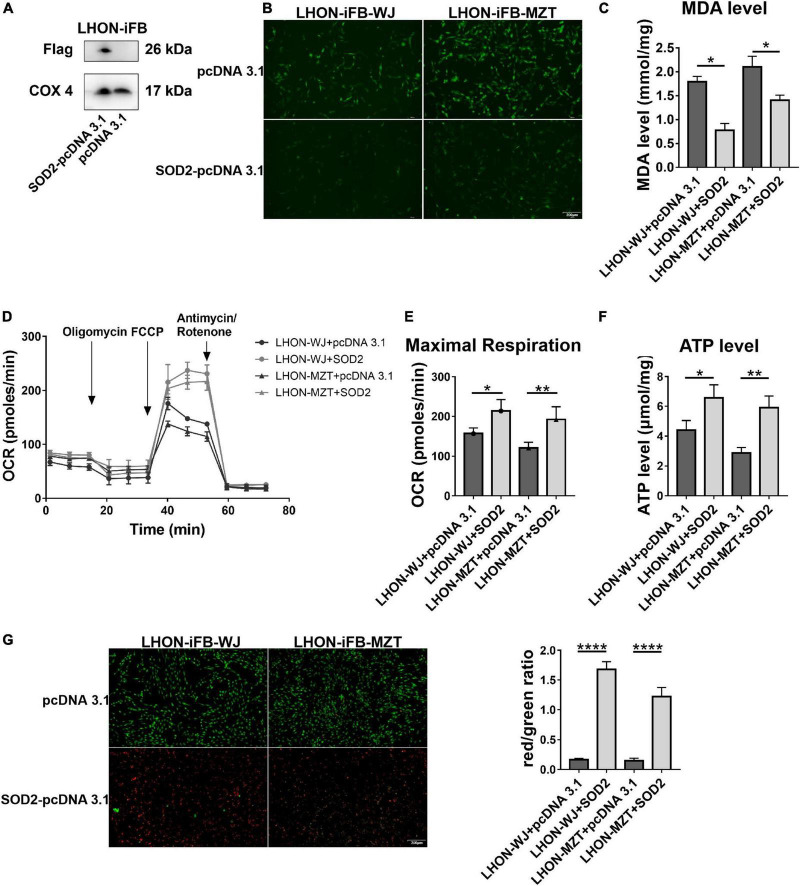
Protective effects of SOD2 on oxidative stress and mitochondrial dysfunction in LHON-iFBs. **(A)** The protein levels of exogenous SOD2 in mitochondria were detected by Western blot. The **(B)** ROS level, **(C)** MDA level, **(D,E)** OCR, and **(F)** ATP level in LHON-iFBs were measured immediately after samples were prepared from each group. **(G)** Representative fluorescence intensity images and red/green ratio of JC-1 fluorescence and from mitochondria in each group. The results (mean ± SD) were statistically significant (*p < 0.05, **p < 0.01, ****p < 0.0001, *n* = 3–5) by using one-way ANOVA with Bonferroni correction, and the assays were performed three times.

Next, we assessed the protective effects of exogenous SOD2. Firstly, we confirmed the overexpression of SOD2 mediated by plasmid (Figure 3A, *n* = 3). Subsequently, we found that compared with cells treated with negative control plasmid, LHON-iFBs transfected with SOD2 showed significantly decreased ROS (Figure 3B, *n* = 4) and MDA level (Figure 3C, *n* = 5), suggesting SOD2 suppressed oxidative stress in LHON fibroblasts.

Mitochondrial energy metabolism analysis showed the OCR, particularly maximal respiration (Figures 3D,E, *n* = 3), and ATP levels (Figure 3F, *n* = 3) were significantly increased in the SOD2 treated group when compared with the negative control group. JC-1 fluorescence revealed that the SOD2 group displayed weaker green fluorescence intensity and stronger red fluorescence intensity than the negative control group, indicating SOD2 ameliorated the decrease of mitochondrial membrane potential (Figure 3G, *n* = 5). SOD2 protected mitochondrial dysfunction in LHON fibroblasts.

**FIGURE 4:**
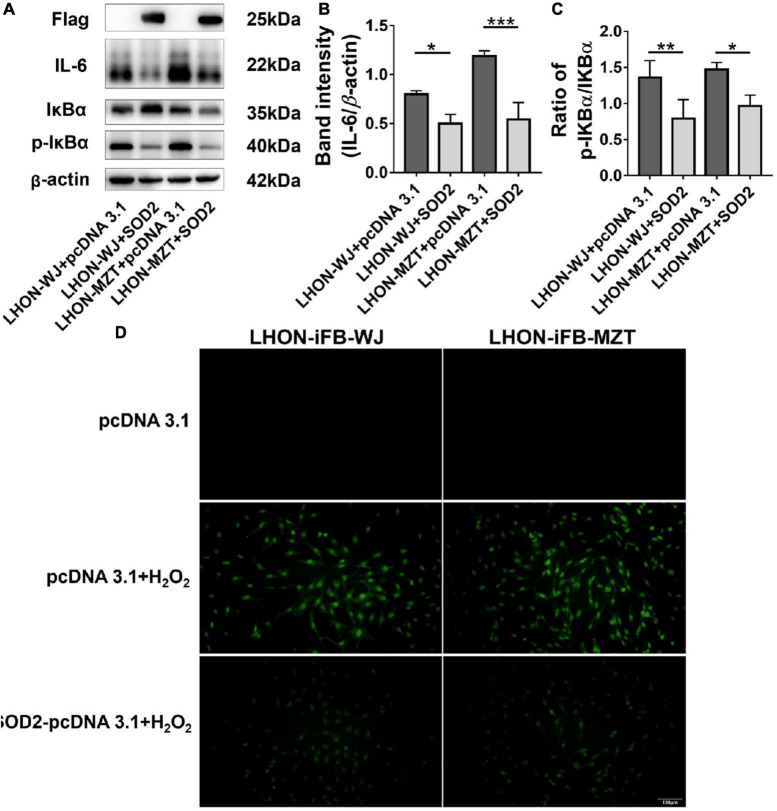
Protective effects of SOD2 on inflammatory response and apoptosis in LHON-iFBs. Western blot showed that **(A,B)** protein levels of IL-6 and **(A,C)** the ratio of p-IκBα to IκBα were inhibited by SOD2 significantly in LHON-iFBs transfected with SOD2 plasmid. **(D)** TUNEL staining demonstrated cell apoptosis in LHON-iFBs transfected with SOD2 plasmid or negative control plasmid for 48 h, respectively, before exposure to hydrogen peroxide for 12 h. The results (mean ± SD) were statistically significant (*p < 0.05, **p < 0.01, ***p < 0.001, *n* = 3–4) by using one-way ANOVA with Bonferroni correction, and the assays were performed three times.

In addition, LHON-iFB group transfected with SOD2 displayed decreased ratio of p-IκBα to IκBα protein and the level of IL-6 (Figures 4A–C, *n* = 4), which indicated that SOD2 downregulated the phosphorylation of IκBα and the secretion of inflammatory cytokines IL-6.

LHON-iFBs were transfected with SOD2 plasmid or negative control plasmid for 48 h, respectively, before exposure to hydrogen peroxide for 12 h. LHON fibroblasts overexpressed with exogenous SOD2 displayed weaker fluorescence of apoptosis marker than the negative control group (Figure 4D, *n* = 3). SOD2 reduced hydrogen peroxide-induced apoptosis in LHON-iFBs.

### Superoxide dismutase 2 prevented hydrogen peroxide induced oxidative stress, mitochondrial dysfunction and apoptosis in human primary retinal pigmental epithelium and SH-SY5Y cells

**FIGURE 5:**
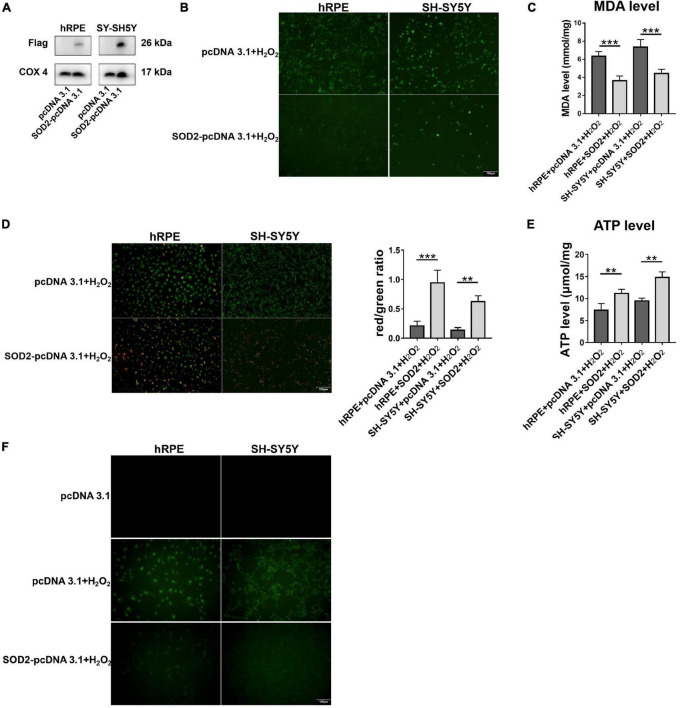
Protective effects of SOD2 on hydrogen peroxide-induced oxidative stress, mitochondrial dysfunction and cell apoptosis in hRPE and SH-SY5Y. **(A)** The protein levels of exogenous SOD2 in mitochondria were detected by Western blot. The **(B)** ROS level and **(C)** MDA level in hRPE and SH-SY5Y were measured immediately after samples were prepared from each group. **(D)** Representative fluorescence intensity images and red/green ratio of JC-1 fluorescence from mitochondria in each group. **(E)** ATP level was detected in hRPE and SH-SY5Y cells. **(F)** TUNEL staining demonstrated cell apoptosis in hRPE and SH-SY5Y. The results (mean ± SD) were statistically significant (**p < 0.01, ***p < 0.001, *n* = 3–5) by using one-way ANOVA with Bonferroni correction, and the assays were performed three times.

To further confirm the protective effects of SOD2 in neurons, we repeated hydrogen peroxide-induced oxidative stress in other two cell models. Primary human retinal pigment epitheliums (hRPE) and human neuroblastoma cells (SH-SY5Y) were transfected with SOD2 or negative control plasmids for 48 h, respectively, before exposure to hydrogen peroxide for 12 h. SOD2 overexpressed substantially in gene transfected hRPE and SH-SY5Y cells (Figure 5A, *n* = 5).

Compared with the control group, SOD2 significantly decreased ROS (Figure 5B, *n* = 3) and MDA levels (Figure 5C, *n* = 5) in hRPE and SH-SY5Y cells treated with hydrogen peroxide.

Then we examined the effects of SOD2 on mitochondrial membrane potential and ATP levels in hRPE and SH-SY5Y cells underwent oxidative stress. In concert with the above findings, weaker green fluorescence intensity and stronger red fluorescence intensity was evident in the SOD2 group than the negative control group, which meant higher membrane potentials (Figure 5D, *n* = 3). Similarly, the level of ATP (Figure 5E, *n* = 4) was higher in the SOD2 treated group. These data indicated that hydrogen peroxide induced mitochondrial dysfunction in hRPE and SH-SY5Y cells was alleviated by exogenous SOD2.

Hydrogen peroxide (400 μM/2 h) increased TUNEL-positive cells as compared with the PBS treated hRPE and SH-SY5Y cells. However, cells transfected with SOD2 exhibited weaker staining (Figure 5F, *n* = 3). SOD2 reduced hydrogen peroxide-induced apoptosis in hRPE and SH-SY5Y cells.

## Discussion

The majority of LHON pathogenic mutations affect a single subunit of mitochondrial complex I (mitochondrial NADH dehydrogenase), causing dysfunction of electron transport chain. Complex I is the largest and most intricate mitochondrial OXPHOS complexes. It is comprised of 45 subunits, 7 (ND-1, −2, −3, −4, −4L, −5, and −6) of which are coded by the mtDNA (Hirst, 2009). Complex I transfers electrons from NADH to ubiquinone, and the energy release is coupled to proton translocations, which contributes to the generation and maintenance of membrane potential (DiMauro et al., 2013). LHON mutations variably affect Complex I, ultimately resulting in energetic deficiency and redox imbalance (Yu-Wai-Man et al., 2011).

In a damaged respiratory chain, electrons overflow and react with molecular oxygen, and eventually lead to the generation of ROS. Excessive ROS production may damage the respiratory subunits and other mitochondrial enzymes, causes lipid membrane peroxidation and apoptosis. These effects lead to cell energy failure and cell death (Yu-Wai-Man et al., 2011; DiMauro et al., 2013).

Recently, Rovcanin et al. (2021) reported increased oxidative stress level in the plasma of LHON patients. Investigations in mouse and transmitochondrial cytoplasmic hybrid (cybrid) model showed similar features resembling LHON, indicating oxidative stress played a critical role in the pathophysiology of LHON (Brown et al., 2000; Lin et al., 2012). On the other hand, previous studies have tested the therapeutic effects in LHON patients and several models. It was suggested that antioxidants including SOD2, coenzyme Q10, idebenone, and EPI-743 could reduce oxidative stress in this condition (Jurkute et al., 2019). In LHON cybrid cell model, estrogens reduced ROS production and apoptosis by activating mitochondrial biosynthesis and improving the metabolic defect (Giordano et al., 2011).

Retinal ganglion cells (RGC) are the main target of LHON, which are not accessible for experiment (Zhang et al., 2010). Various alternative strategies have been developed for LHON research due to the lack of access to RGCs. At present, iPS-RGC cells are a favored emerging cell model (Peron et al., 2020; Harvey et al., 2022; Singh et al., 2022). Fibroblasts have been induced into RGC-like cells by reprogramming of factors stimulation (Peron et al., 2020) or Sendai virus infection (Wu et al., 2018) and these cells present mitochondrial damage phenotype (Wu et al., 2018; Yang et al., 2020). This strategy allows us to obtain a cell model similar to optic nerve tissue for study. However, some limitations still exist. For example, the clone number was insufficient after differentiation due to immature technology (Peron et al., 2021), and mutated mtDNA might be missed in partial RGCs differentiated from iPSC{Peron, 2021 #79}. Skin-derived fibroblasts are a proven alternative cell model (Jankauskaite et al., 2017). As cells do not undergo differentiation processes, the genetic background is more stable. And fibroblasts have the advantages of easy obtaining and culture. Although fibroblasts cannot used for the study of axon degeneration and neural signaling, multiple studies demonstrated that they can be used as a valuable strategy for studying mitochondrial impairment in neurological disorders (Olesen et al., 2022). In addition, our previous study (Yao et al., 2022) and the current manuscript have proved that fibroblasts could reflect some cellular phenotypes of RGC cells with LHON, such as energy metabolism, mitochondrial dysfunction, and oxidative stress, which was consistent with iPSC. Therefore, in this study, we conducted LHON patients’ skin derived fibroblasts for the study of mitochondria function and inflammation. Nevertheless, iPS cell is still a cell model of significant potential in the future with its unique advantages.

We constructed immortalized skin fibroblasts LHON-iFB derived from severe LHON patients. These cells presented mitochondrial dysfunction, oxidative stress and NF-κB associated inflammatory response. We further showed that SOD2 ameliorated oxidative stress, mitochondrial dysfunction, inflammatory response, and apoptosis in the LHON-iFBs. To further prove the protective effects of SOD2, we constructed additional oxidative stress cell models. Overexpression of SOD2 alleviated oxidative stress, mitochondrial dysfunction and apoptosis in hRPE and SH-SY5Y cells exposed to hydrogen peroxide. These data further supported the protective effects of SOD2.

A large number of studies have demonstrated that inflammatory response plays a critical role in neurodegenerative disorders (Glass et al., 2010; van Horssen et al., 2019). However, reports that inflammatory response is involved in the pathophysiology of LHON are limited. We observed that the ratio of p-IκBα to IκBα protein and the level of IL-6 increased in LHON-iFBs. These results could be direct evidence that NF-κB associated inflammatory response were involved in LHON. However, the detailed role of inflammatory response in LHON is still unknown. As a possible interpretation, inflammatory responses might be a consequence of mitochondrial dysfunction. Released during severe injury induced by stress or infection, macromolecules damage-associated molecular patterns (DAMPs) are capable of eliciting a strong local inflammatory response (Chen and Nunez, 2010). Mitochondrion is a source of DAMPs. As such, mitochondrial dysfunction results in the release of DAMPs within the cytosol or in the extracellular environment thereby eliciting innate immune activation (Tait and Green, 2012). We speculate that energetic deficiency and redox imbalance increases mitochondrial membrane permeability and induces the release of mitochondrial components, such as mtDNA or cardiolipin, which in turn elicited inflammatory response. Inflammatory response seen in LHON models is an interesting phenomenon. Clearly, extensive experiments are necessary to further verify it and its underlying mechanisms.

In conclusion, skin derived LHON-iFB presented some crucial pathophysiologic features of LHON including mitochondrial dysfunction, oxidative stress, inflammatory response and susceptibility to insult. Mitochondria-targeted antioxidase SOD2 ameliorated mitochondrial dysfunction by inhibiting oxidative stress and cell death. Our data further support the notion that augment of exogenous SOD2 may be a promising therapy for the treatment of LHON (Qi et al., 2007).

## Conclusion

The results showed LHON-iFB presented mitochondrial dysfunction and increased ROS, suggesting the skin fibroblasts derived from the patients may be a valuable model to study LHON. Exogenous SOD2 ameliorated mitochondrial dysfunction by inhibiting oxidative stress and preventing oxidative stress-induced cell death. The protective effects might be associated with reducing inflammatory response and inhibiting NF-κB signaling. The data further support exogenous SOD2 may be a promising therapy for the treatment of LHON.

## Data availability statement

The original contributions presented in this study are included in the article/supplementary material, further inquiries can be directed to the corresponding author/s.

## Ethics statement

The studies involving human participants were reviewed and approved by the Ethics Committee of Henan Eye Hospital [IRB approval number: HNEECKY-2019 (12)]. The patients/participants provided their written informed consent to participate in this study. Written informed consent was obtained from the individual(s) for the publication of any potentially identifiable images or data included in this article.

## Author contributions

BL and QZ designed the study and wrote the manuscript. QZ and SY performed the experiments and analyzed the data. LL helped in performing the experiments. MY, QG, and YL collected patients’ information, prepared the fibroblasts, and RPE cells. BL revised the manuscript, supervised the study, and provided financial support. All authors read and approved the final manuscript.

## Abbreviations

LHON: Leber’s hereditary optic neuropathy
SOD: mitochondrial superoxide dismutase
ROS: reactive oxygen species
mtDNA: mitochondrial DNA
iFB: fibroblasts
hRPE: human primary retinal pigmental epithelium
OCR: oxygen consumption rate
ATP: adenosine triphosphate
MDA: malondialdehyde
TUNEL: terminal-deoxynucleoitidyl transferase mediated nick end labeling
OXPHOS: oxidative phosphorylation
CDS: coding sequence
FCCP: carbonyl cyanide 4-(trifluoromethoxy) phenylhydrazone
MMP: mitochondrial membrane potential
TBA: thiobarbituric acid
PBS: Phosphate Buffered Saline
PMSF: phenylmethylsulfonyl fluoride
WB: western blot
IP: immunoprecipitation
BCA: bicinchoninic acid
PVDF: polyvinylidene difluoride
TBST: tris buffered saline with tween-20.
